# Anticyanobacterial effect of l-lysine on *Microcystis aeruginosa*

**DOI:** 10.1039/c8ra00434j

**Published:** 2018-06-13

**Authors:** Lili Tian, Meng Chen, Chongyang Ren, Yiying Wang, Li Li

**Affiliations:** Shandong Provincial Key Laboratory of Water Pollution Control and Resource Reuse, School of Environmental Science and Engineering, Shandong University Jinan China lili@sdu.edu.cn; Shandong Provincial Engineering Center on Environmental Science and Technology Jinan China

## Abstract

Cyanobacterial blooms can cause serious environmental problems and threaten aquatic organisms and human health. It is therefore essential to effectively control cyanobacterial blooms in aquatic ecosystems. In the present study, the anticyanobacterial effect of l-lysine on *Microcystis aeruginosa* was examined. The results showed that the growth of *M. aeruginosa* (>90%) was effectively inhibited by l-lysine at dosages of 5.0, 6.5, and 8.0 mg L^−1^ after 3 d treatment. The content of superoxide anion radicals, MDA content and SOD activity in *M. aeruginosa* cells increased after 1 d of treatment with l-lysine (3.0, 5.0, 6.5, and 8.0 mg L^−1^), revealing that l-lysine induced oxidative stress in the cyanobacterial cells. The chlorophyll-*a* and protein contents in *M. aeruginosa* treated with l-lysine (3.0, 5.0, 6.5, and 8.0 mg L^−1^) decreased after 2 d, indicating damage of the photosynthetic system by l-lysine treatment. Additionally, the production of exopolysaccharide by *M. aeruginosa* also increased and the expression of polysaccharide synthesis genes was upregulated by 3.0 mg L^−1^l-lysine after 3 d of treatment. In response to the algicidal effects of l-Lysine, *M. aeruginosa* upregulated exopolysaccharide synthesis. Electron microscopic observations demonstrated that the cell membrane of *M. aeruginosa* was broken down during treatment with l-lysine (≥3.0 mg L^−1^). Our results revealed that the effects of l-lysine on *M. aeruginosa* cells were comprehensive, and l-lysine is therefore an efficient anticyanobacterial reagent.

## Abstract 2

l-lysine had an anticyanobacterial effect on *Microcystis aeruginosa*, which involved growth inhibition, physiological and metabolic disturbance, and cell membrane damage.

## Graphical abstract

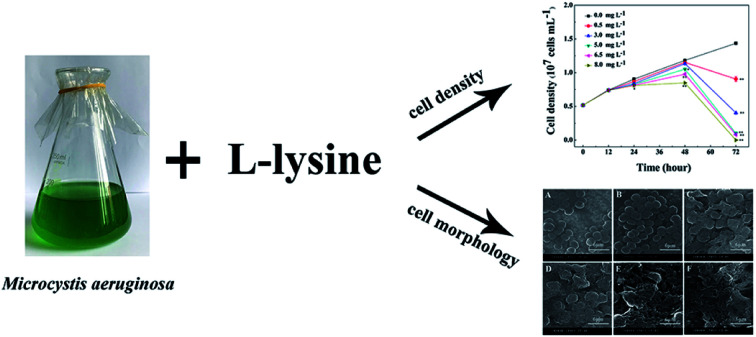

## Introduction

*Microcystis* is one of the well-known types of bloom-forming cyanobacteria in freshwater worldwide.^1^ Seasonal massive growth of cyanobacteria has threatened drinking water supplies, recreation, tourism, and fisheries.^2^ Moreover, many strains of *Microcystis* species synthesize harmful monocyclic hepatotoxins known as microcystins that adversely affect water quality, aquatic organisms and human health.^3^ Therefore, effective strategies to control and eliminate cyanobacterial blooms are urgently needed.

Current methods of controlling *Microcystis* include the application of chemical treatment, physical techniques, or physical/chemical combination technologies, such as chlorination,^4^ UV-radiation,^5^ and ultrasound-coupled TiO_2_.^6^ Chemical methods remain the predominant technique for killing algae, and this technology has been developed to be direct, quick, effective, and convenient to operate. These approaches, although effective for the acute reduction of harmful algae, are costly or may produce secondary pollution.

Biological methods are an attractive alternative. Algicidal bacteria are an effective natural resource with which to control *Microcystis*, and include *Bacillus* sp.,^7^*Citrobacter* sp.,^8^*Trichoderma citrinoviride*,^9^*Acinetobacter guillouiae*,^10^*Pseudomonas aeruginosa*,^11^ and *Aeromonas* sp.^12^ These algicidal bacteria exert an inhibitory effect on *Microcystis* in direct or, more well-known, indirect ways.^7,10,12^ However, due to unreliable performance under various environmental conditions, the successful application of *Microcystis*-killing microbiota in nature is yet to be fully confirmed.^13^

Antialgal natural materials have attracted increasing attention, which have the advantage of operating *via* both biological and chemical methods to control algae. Previously, researchers demonstrated that a bioactive substance, l-lysine, has a significant algicidal effect on *Microcystis* species.^14,15^ It is well known that all fish species cannot survive without lysine.^16^ A recent study demonstrated the specific effect of l-lysine on cyanobacteria, to which benign chlorophytes showed low sensitivity.^17^ Therefore, l-lysine is a promising candidate for the control of cyanobacteria in aqueous ecosystems. However, the mechanism of action of l-lysine against *Microcystis* cells remained unknown, which limited its potential application in the environment. The purpose of this study was to investigate the anticyanobacterial mechanism of l-lysine. To achieve this, we examined the effect of l-lysine on *Microcystis aeruginosa* in terms of cell growth, physiological properties, and cell morphology.

## Materials and methods

### Culture and l-lysine treatment of cyanobacteria

*M. aeruginosa* NaRes975, a bloom-forming strain isolated from a reservoir, was obtained from the Institute of Hydrobiology, Chinese Academy of Science (Wuhan, China). The cyanobacterium was cultivated in BG11 medium at 25 ± 1 °C under a cycle of 12 h light: 12 h dark with a light intensity of 50 μmol photons m^−2^ s^−1^.

*M. aeruginosa* was cultured to exponential phase, then collected and re-suspended in fresh BG11 medium with an initial density of 5.2 × 10^6^ cells per mL. To test the effect of l-lysine on the cyanobacterium, lysine was added to the cell suspension (200 mL) to a final concentration of 0.5, 3.0, 5.0, 6.5, and 8.0 mg L^−1^, respectively. All of the tests were conducted in triplicate, and the same cell suspensions were prepared without lysine as a control. The cell densities were measured by counting cells in hemocytometers using a light microscope.^18^

The growth inhibition ratio (%) was evaluated using the following formula: (1 − *N*_t_/*N*_0_) × 100, where *N*_t_ (treatment) and *N*_0_ (control) are the cell densities of *M. aeruginosa* with and without l-lysine treatment, respectively.

### Physiological indicators measurement

Chlorophyll-*a* (Chl-*a*) was measured every 24 h by methanol extraction^19,20^ and analysis of the Chl-*a* autofluorescence of cell suspensions using a Nikon TE2000 microscope (Japan) after photophobic adaptation for 10 min.^19^ To detect superoxide anion radical (O_2_^−^), malondialdehyde (MDA), superoxide dismutase (SOD), and intracellular protein, *M. aeruginosa* cells were homogenized by ultrasound, the cell-free enzyme supernatant was prepared. The content of O_2_^−^ was measured by using a spectrophotometric method.^21^ The content of O_2_^−^ is indicated by the ratio of absorption at 530 nm. The MDA content was measured according to the thiobarbituric acid method.^22^ SOD activity was measured by self-oxidation of pyrogallic acid.^23^ The O_2_^−^ content, MDA content, SOD activity in the cells treated with l-lysine were evaluated by the ratio of measurement in experimental group to that in the control group. The ratios of OD_530_, MDA and SOD were calculated using the following formula: *R*_t_/*R*_0_, where *R*_t_ (treatment) and *R*_0_ (control) represent the ratio of measurement value to the corresponding cell density in the experimental and control groups, respectively. The intracellular protein content was tested using a Folin–Lowry kit (Dingguo Changsheng Biotechnology Co. Ltd., Beijing, China) using bovine serum albumin as a standard.

### Acidic polysaccharide analysis

Acidic polysaccharides exposed on cell surfaces were analyzed by a method of Alcian blue GX staining. Briefly, cyanobacterial cells were harvested and washed with distilled water, then incubated for 30 min with 3% acetic acid at ambient temperature. Following this, the cells were collected and resuspended with Alcian blue reagent (0.33% Alcian blue GX in 3% acetic acid, pH 2.5) for 10 min. The excess dye was removed with distilled water, and the stained cells were observed by light microscopy.^24^

### RNA extraction, reverse transcription, and quantitative PCR analysis

Total RNA was extracted using a Spin Column Bacterial Total RNA Purification Kit (Sangon Biotech, Shanghai, China). The RNA concentration and purity were evaluated spectrophotometrically using a NanoDrop 2000 spectrophotometer, and RNA integrity was assessed by 1% formaldehyde agarose gel electrophoresis. About 1.7–4.4 μg of total RNA was used for cDNA synthesis with random hexamer primers using a RevertAid First Stand cDNA Synthesis Kit (Thermo Fisher Scientific, Waltham, MA, USA). The cDNA was stored at −70 °C for further use.

Two polysaccharide synthesis genes were selected to investigate their relative changes in expression during l-lysine treatment. Relative quantification was performed using the housekeeping gene encoding a ribosomal protein (RP-S11) as a calibrator. The primer pairs used to amplify the two target genes and the housekeeping gene are shown in Table 1. Quantitative real time PCR (RT-PCR) was conducted on a BioRad iCycler (BioRad, Hercules, CA, USA). Each qPCR reaction was performed in a 20 μL reaction mixture containing diluted cDNA, 500 nM of each primer, and 10 μL SYBR Green Mastermix (BioRad). The qPCR was performed under the following conditions: an initial denaturation step at 95 °C for 30 s, followed by 40 cycles of 95 °C for 5 s and 56 °C for 10 s. All qPCR assays were run in triplicate for different samples. Each PCR run included a no-template control well containing water instead of cDNA as a negative control for each gene. The copy number was determined using threshold cycle (*C*_t_) values. The relative quantities of the target gene were determined using the 2^−ΔΔ*C*_t_^ method.^25^ The PCR products were detected by agarose gel electrophoresis and sequencing analysis.

**Table 1:** Primers used in this study

Target gene accession no. (start…stop)	Predicted products	Primer sequences (5′–3′)	Amplicon length (bp)
MOLN01000044.1 (120…410), *bcsA*	Cellulose synthase	F: TGCTCTCAAACAAACCCAATGT	131
		R: TTTGGGGAGTTTGCACTAAGGC	
MOLN01000086.1 (9700…10968)	Dolichol-phosphate mannosyltransferase (DPM1)	F: CCAAGAAAATCATCGGATTCGC	108
		R: ACTACTGCCACTGCATCACC	
MOLN01000055.1 (1456…1848)	Small subunit ribosomal protein S11 (RP-S11)	F: GGTCTATCGCGTTCCCAAGA	146
		R: CTCCCAGCGTCTCAAATCCC	

### Electron microscope observations

To observe the changes in morphological features of *M. aeruginosa* following l-lysine treatment, cells were collected after a 3 d treatment with l-lysine and observed under a scanning electron microscope (SEM). Samples were prepared using the method modified from Ahmadjian *et al.*^26^ The cells were washed twice with 0.1 M PBS buffer (pH 7.2), fixed in 2.5% glutaraldehyde at 4 °C for 24 h, and then post-fixed in 1% osmium tetroxide at 4 °C for 60 min, after which they were rinsed with PBS buffer. Specimens were then dehydrated using an acetonitrile series (50, 75, 95, and 100%) and dried with a Critical-Point Dryer (HCP-2, Hitachi Electronic Instruments, Tokyo, Japan). Finally, specimens were sputter-coated with platinum and examined under an H-8010 SEM (Hitachi, Japan).

### Statistical analysis

Independent triplicate assays were performed and all of the data are expressed as the mean ± standard deviation. Data were analyzed by one-way analysis of variance (ANOVA) using SPSS version 22.0 software. A significant difference was considered at *p* < 0.05.

### Genomic sequence accession number of *M. aeruginosa*

The draft genome sequence of *M. aeruginosa* NaRes975 was deposited in the DDBJ/EMBL/GenBank database under accession number MOLN00000000.

## Results and discussion

### Anticyanobacterial effects of l-lysine on *M. aeruginosa*

**Fig. 1:**
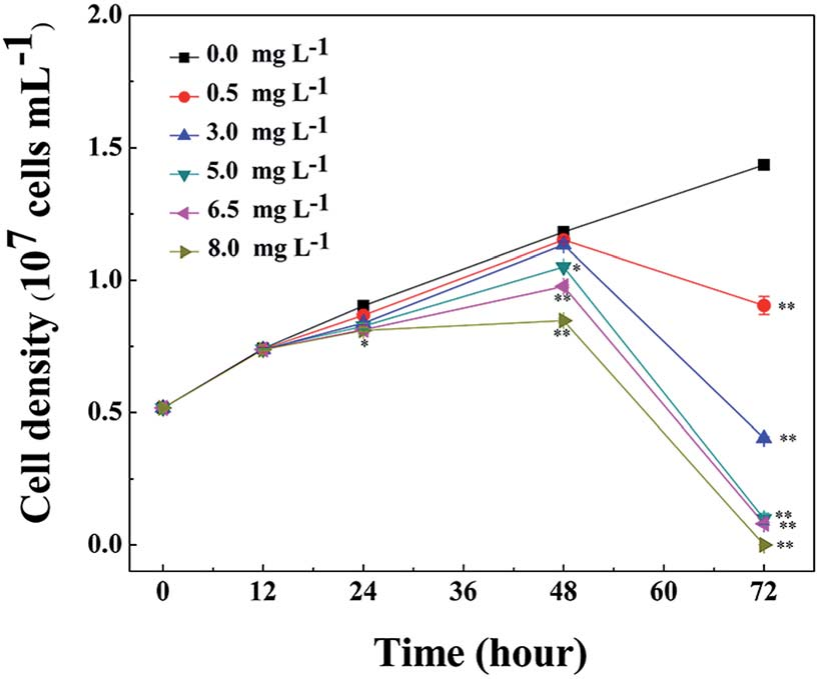
Cell densities of *M. aeruginosa* during treatment with 0.5, 3.0, 5.0, 6.5, and 8.0 mg L^−1^l-lysine. *M. aeruginosa* without l-lysine treatment served as a control. Data presented are expressed as the mean ± standard deviation. Significant differences between the test and control groups are indicated by asterisks, * when *p* < 0.05, and ** when *p* < 0.01.

The growth inhibition effect of l-lysine on *M. aeruginosa* was tested over a concentration ranging from 0.5 mg L^−1^ to 8.0 mg L^−1^ and the results are shown in Fig. 1. Compared with the control group, l-lysine exhibited a significant inhibitory effect on *M. aeruginosa* after treatment for 24 h. However, the effects on *M. aeruginosa* following treatment with different concentrations of l-lysine over 12 h were indistinguishable. The inhibitory effect was enhanced with increased l-lysine concentration and the prolongation of treatment time. After treatment with l-lysine at concentrations ranging from 0.5 mg L^−1^ to 8.0 mg L^−1^ for 72 h, an inhibition ratio of 36.9% to approximate 100% was achieved. In a previous study, dissolved free amino acids, such as alanine, arginine, and leucine enhanced the growth of *M. aeruginosa*, by contrast, lysine inhibited cyanobacterial growth.^27^ Recently, it was reported that l-valine also showed an inhibitory effect on *Microcystis* growth. However, *M. aeruginosa* was more liable to recover its growth after treatment with l-valine than after treatment with l-lysine.^22^ These results indicated that l-lysine has the potential to be applied for the control of *M. aeruginosa*.

Antialgal natural materials are promising in natural aqueous ecosystem. Comparing with algicidal secondary metabolites from different organisms, amino acids are more moderate and safer to the vast majority of species in aqueous ecosystem. However, the effectiveness of amino acids on harmful algae needs to be examined and compared with other algicides. For example, the dosage for treatment and the inhibition rates are decisive factors for the applicability of an antialgal material. In this sense, l-lysine has competition advantage as algicides.^28^ On the other hand, due to the important roles played by amino acids in cells, the antialgal mechanisms of l-lysine are complicated, the antialgal mechanisms of l-lysine remain unclear.

### Influences of l-lysine on O_2_^−^content, MDA content and SOD activity of *M. aeruginosa*

O_2_^−^ is the primal oxygen radical in cells, the MDA content and SOD activity are efficient indicators of the physiological response of cyanobacteria to algicides.^11,21,22,29^ As a precursor of active free radicals in cells, the increase of O_2_^−^ content causes oxidative stress. As a representative product of peroxidation, MDA is commonly used as a marker of lipid peroxidation under stress conditions.^11,21,22,29^ The presence of O_2_^−^ in cells may initiate a series of harmful reactions, such as lipid peroxidation, which leads to an accumulation of MDA in the cells. As an important antioxidase, SOD activity can reflect the response of enzymatic antioxidant defense systems to relieve environmental stress, therefore indicating the severity of the environmental stress.

**Fig. 2:**
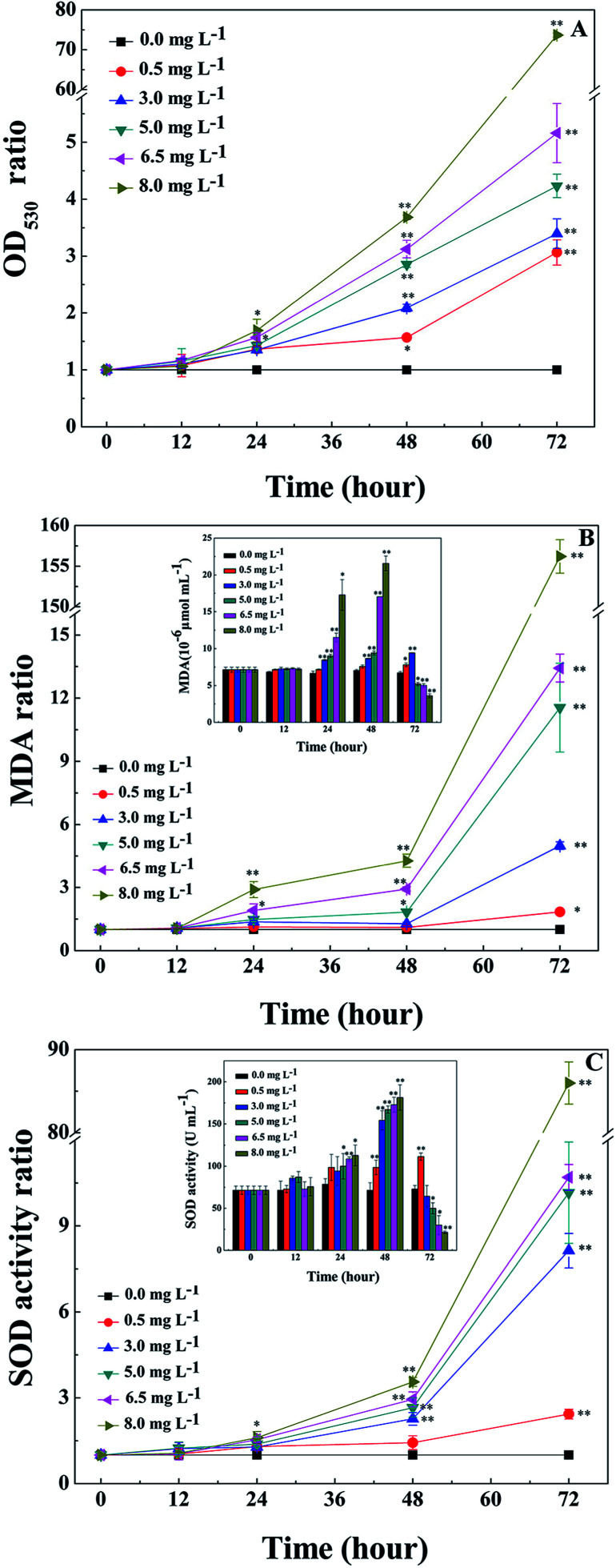
Effects of l-lysine on the O_2_^−^ content, MDA content and SOD activity of *M. aeruginosa* cells. Cells were treated with 0.5, 3.0, 5.0, 6.5, and 8.0 mg L^−1^l-lysine. *M. aeruginosa* without l-lysine treatment served as a control. Data presented are expressed as the mean ± standard deviation. Significant differences between the test and control groups are indicated by asterisks, * when *p* < 0.05, and ** when *p* < 0.01.

The O_2_^−^ contents of cyanobacterial cells were measured during treatment with l-lysine. As shown in Fig. 2A, the O_2_^−^ contents increased significantly after 1 d when treated with l-lysine (*p* < 0.01 or *p* < 0.05). The O_2_^−^ contents increased with the increase of l-lysine level and the prolongation of treatment time. The changes in MDA contents of cyanobacterial cells are shown in Fig. 2B. The MDA content was relatively stable following treatment with 0.5 mg L^−1^l-lysine within 48 h, similar to control levels. Whereas the MDA contents in the other treatment groups increased along with increased l-lysine levels after 12 h. It was noticed that the total MDA contents in the treatment group decreased after 48 h along with the sharp decline of cell density. However, the ratio of MDA in the treatment groups to that in the control group indicated the significant increase of MDA contents in the cyanobacterial cells during treatment with l-lysine (*p* < 0.01 or *p* < 0.05). The increase in MDA content after treatment with l-lysine indicated the occurrence of lipid peroxidation, which correlated with the l-lysine level. The effect of l-lysine on the SOD activity of *M. aeruginosa* is shown in Fig. 2C. Similarly, the SOD activity of cyanobacterial cells increased significantly during treatment with l-lysine comparing with the untreated control (*p* < 0.01 or *p* < 0.05). The SOD activity in the *M. aeruginosa* cells increased under the oxidative stress induced by l-lysine, indicating that the oxidative stress trigged cellular defense responses. In a recent study, O_2_^–^ was detected to be induced dramatically in *M. aeruginosa* during treatment with l-valine, along with an increase in the MDA content and SOD activity.^22^ It was suggested that some exogenous amino acids, including l-lysine and l-valine, might induce serious oxidative stress in cyanobacterial cells, which could destroy the cells directly.

### Chl-*a* and protein variation by l-lysine treatment

**Fig. 3:**
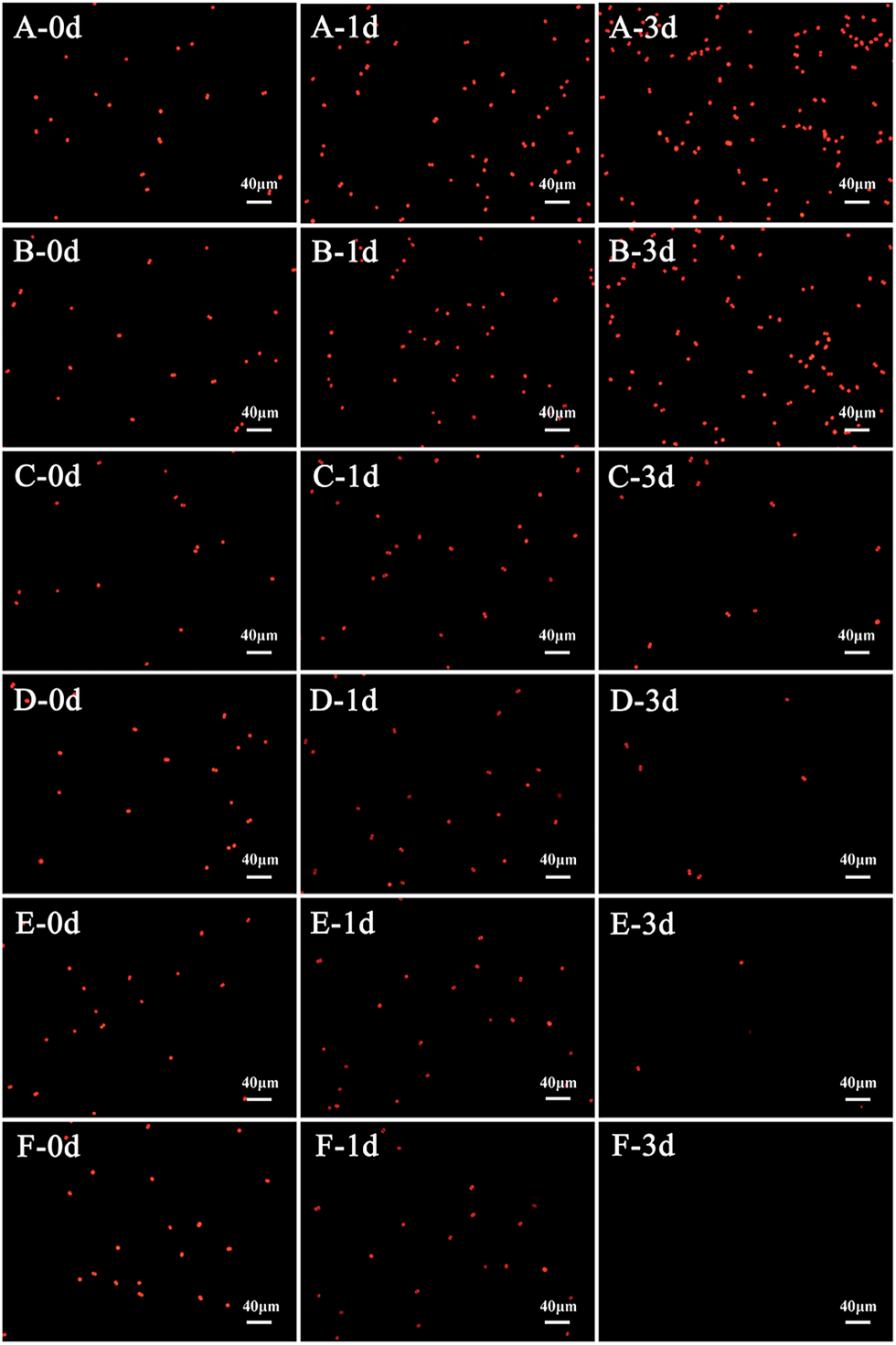
Chlorophyll-*a* autofluorescence images of *M. aeruginosa* cells during treatment with different dosages of l-lysine. A control without treatment is shown in (A); 3 d treatments with l-lysine at 0.5 mg L^−1^, 3.0 mg L^−1^, 5.0 mg L^−1^, 6.5 mg L^−1^, and 8.0 mg L^−1^ are shown in (B) to (F), respectively.

During the process of l-lysine treatment, the color of *M. aeruginosa* gradually turned yellow compared with the control group, with the exception of the group treated with 0.5 mg L^−1^l-lysine. This indicated that the exogenous l-lysine influenced the pigments of the cell. Chl-*a* autofluorescence can be viewed as a probe for photosynthesis *in vivo* and reflects the correct functioning of photosystem II.^30^ Cell activity and density can be reflected by red fluorescence. Chl-*a* autofluorescence of cyanobacterial cells was monitored during treatment with different concentrations of l-lysine (Fig. 3). It was shown that 0.5 mg L^−1^ of l-lysine caused no significant change in autofluorescence of *M. aeruginosa* cells after 3 d of treatment compared with the control. While the autofluorescence of *M. aeruginosa* cells in the other test groups (l-lysine dosage > 0.5 mg L^−1^) decreased with increased l-lysine dosage after treatment for 3 d, and even disappeared with 8.0 mg L^−1^l-lysine.

**Fig. 4:**
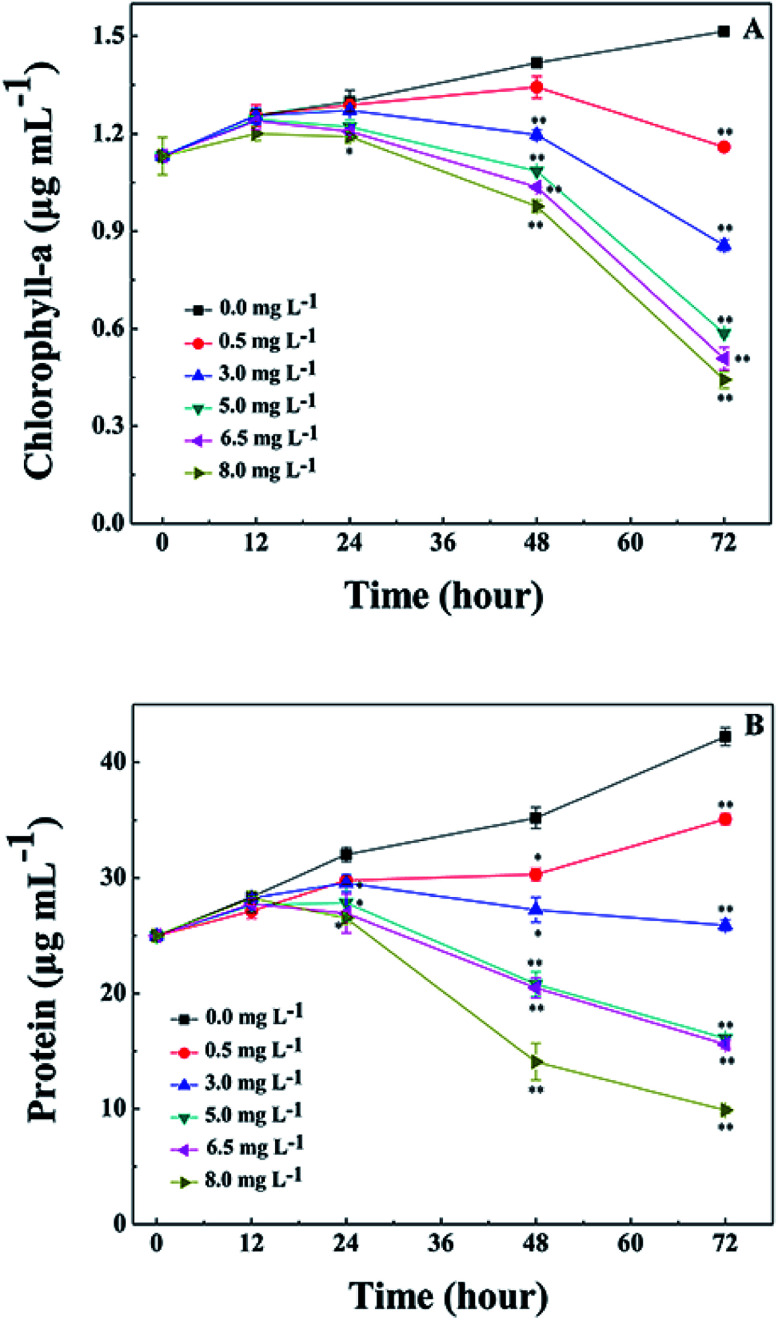
Effects of l-lysine on Chl-*a* and protein levels in *M. aeruginosa* cells. Cells were treated with 0.5, 3.0, 5.0, 6.5, and 8.0 mg L^−1^l-lysine. Cells not treated with l-lysine served as a control. Data presented are expressed as the mean ± standard deviation. Significant differences between the test and control groups are indicated by asterisks, *when *p* < 0.05, and **when *p* < 0.01.

The Chl-*a* content of *M. aeruginosa* was also detected during treatment with l-lysine. As shown in Fig. 4A, l-lysine exerted a significant effect on the Chl-*a* contents during the 3 d treatment. Whereas the Chl-*a* level in the control group increased with time, the Chl-*a* contents in the treatment groups (l-lysine at 0.5, 3.0, 5.0, 6.5, and 8.0 mg L^−1^) decreased after 2 d. The protein contents were also influenced by l-lysine (Fig. 4B). The pattern of changes was similar to that detected for Chl-*a* contents with increasing l-lysine concentration and prolonged treatment time. Zimba and colleagues demonstrated that l-lysine inhibited Chl-*a* accumulation by *M. aeruginosa*, and that concentrations >1.0 mg L^−1^ were required for inhibition.^17^ Zimba and colleagues suggested that the inhibitory effect of l-lysine on Chl-*a* accumulation in *M. aeruginosa* might be associated with the enzymatic process.^17^ It is known that oxygen radical has the potential to attack biological macromolecules, *e.g.*, proteins, lipids and nucleotides. And it can attack photosynthetic pigments such as Chl-*a*.^31^ Thus contents of Chl-*a* and protein decreased along with the promotion of O_2_^−^ content induced by increasing l-lysine. On the other hand, reactive oxygen species can damage or modify essential proteins and lipid components present in the thylakoid membrane, which lead to the disruption of the photosynthetic electron transport chain, and what is more lead to the additional formation of oxygen radical and enhanced oxidative stress.^31^ These results indicated that exogenous l-lysine could damage the photosynthetic system in *M. aeruginosa*.

### Effects of l-lysine on polysaccharide production by *M. aeruginosa*

**Fig. 5:**
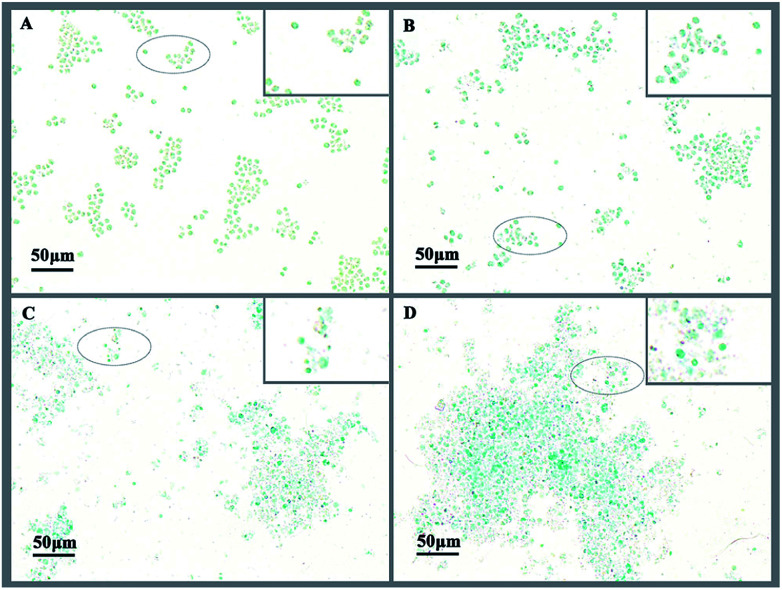
*M. aeruginosa* cells stained with Alcian blue after treatment with l-lysine. A control without treatment is shown in (A); cells treated for 3 d with l-lysine at dosages of 0.5, 3.0, and 5.0 mg L^−1^ are shown in (B) to (D), respectively.

Exopolysaccharides of cyanobacteria, including attached polysaccharide and released polysaccharide, have been implicated in a variety of stress conditions imposed by hazardous stimuli in the environment.^32^ To indicate the variation of exopolysaccharides, acidic polysaccharides adhered to cell wall of *M. aeruginosa* were stained with Alcian blue GX after 3 d treatment with l-lysine. As shown in Fig. 5A, there were no acidic polysaccharides attached to the surface of cyanobacterial cells in the absence of treatment (Fig. 5A). However, the attached acidic polysaccharides increased with increased l-lysine dosage, as shown by the deeper cyan-stained cells in Fig. 5B–D. It should be noted that cells debris of *M. aeruginosa* became evident with increased l-lysine dosage. These results indicated that l-lysine might enhance the synthesis of polysaccharides.

**Fig. 6:**
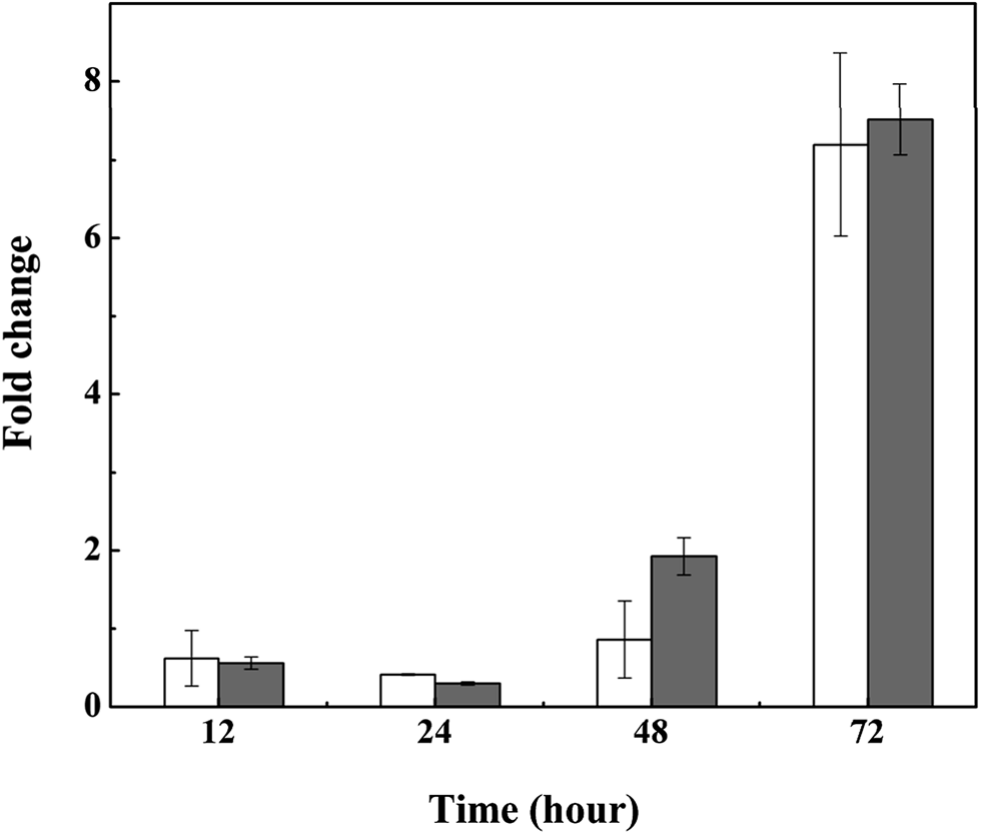
Relative transcriptional activity of polysaccharide synthesis genes in *M. aeruginosa* treated with 3.0 mg L^−1^l-lysine. Cellulose synthase and dolichol-phosphate mannosyltransferase are illustrated by white and gray columns, respectively.

We also tested the transcriptional activity of the polysaccharide synthesis genes of *M. aeruginosa* in the presence of 3.0 mg L^−1^l-lysine. According to the genome sequence of *M. aeruginosa*, the transcriptional activities of two predicted genes involved in polysaccharide synthesis were detected by qPCR. As shown in Fig. 6, the transcriptional activity of these two genes was upregulated in response to l-lysine stress, especially after treatment for 72 h. They showed >7-fold upregulation, indicating that l-lysine influenced polysaccharide synthesis by transcriptional regulation. Similarly, a previous study indicated that the production of exopolysaccharides was enhanced by salt stress in *Synechocystis* strains.^33^ It also reported that Cr(vi) and Cd(ii) were important stress factors that caused the increase of exopolysaccharide production in cyanobacteria.^34,35^ Furthermore, it has been shown that environmental stresses, such as pH and nitrogen concentration, also influenced the production of extracellular carbohydrates in algal cells.^36,37^ It was proposed that the exopolysaccharides produced by various microbes have a strong scavenging ability toward various reactive oxygen species including superoxide and hydroxyl radicals.^38^ Thus, along with the variation in MDA content and SOD activity, the increase in exopolysaccharide production may be another strategy employed by *M. aeruginosa* cells to resist the damage caused by l-lysine, and the mechanism for this metabolic process was found to be based on transcriptional regulation. However, due to the acute algicidal effect of l-lysine, the promotion of exopolysaccharide production did not counteract the disruption of the cells.

### Effects of l-lysine on the morphology of *M. aeruginosa*

**Fig. 7:**
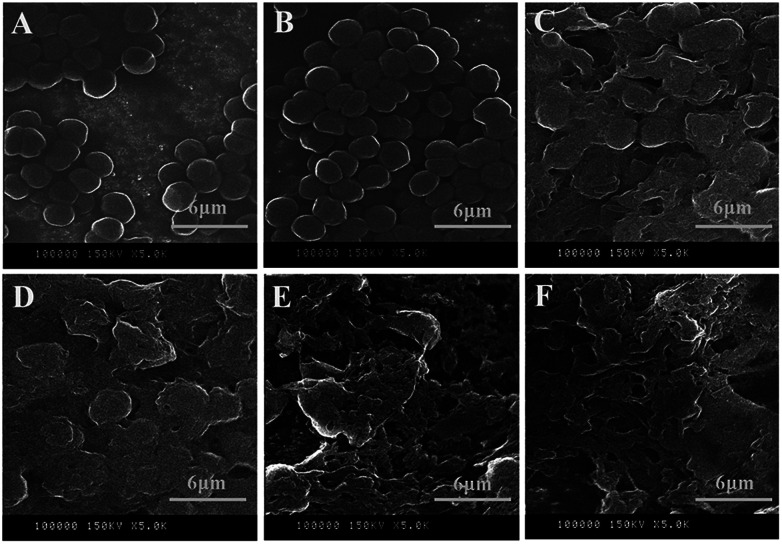
SEM images of *M. aeruginosa* treated with different dosages of l-lysine. A control without treatment is shown in (A); cells treated with l-lysine for 3 d with dosages of 0.5, 3.0, 5.0, 6.5, and 8.0 mg L^−1^ are shown in (B) to (F), respectively.

SEM observations provided information on cell morphology changes and the integrity of cell membranes following l-lysine treatment. Overall, l-lysine inflicted severe damage to *M. aeruginosa* cells. In the absence of l-lysine, *M. aeruginosa* cells were spherical and the surfaces were smooth (Fig. 7A). With 0.5 mg L^−1^l-lysine treatment, the morphology of cells was similar to that of the control samples, which indicated that low concentrations (0.5 mg L^−1^) of l-lysine had little effect on the integrity of the cell membrane (Fig. 7B). However, cyanobacterial cells were destroyed with ≥3.0 mg L^−1^l-lysine treatment after 3 d (Fig. 7C–F). The anticyanobacterial effect of l-lysine on cell morphology appeared to correlate to its dosage after 3 d treatment. Particularly following treatment with dosages of 6.5 mg L^−1^ and 8.0 mg L^−1^l-lysine, no intact cells were detectable and only cell debris remained. l-lysine was therefore capable of completely destroying cyanobacterial cells. This result agreed with a previous study that reported that lysine was responsible for severe damage of the cell wall of *Microcystis*.^15^

Consistent with the results in Fig. 2, l-lysine might induce serious oxidative stress in cyanobacterial cells, leading to membrane lipid peroxidation and destruction of the cell membrane. It was revealed that the membrane of *M. aeruginosa* cells was broken down during treatment with l-lysine, which was followed by cell death as shown in Fig. 1. This may be one of the lethal effects of l-lysine.

## Conclusions

l-lysine could efficiently inhibit the growth of *M. aeruginosa* at dosages above 5.0 mg L^−1^ after 3 d treatment. The effect of l-lysine on *M. aeruginosa* cells was comprehensive. l-lysine (≥3.0 mg L^−1^) could induce oxidative stress and cell membrane damage, as indicated by measurement of the O_2_^−^ content, MDA content and SOD activity and electron microscopy observations. l-lysine also affected the photosynthetic system at different dosages (0.5–8.0 mg L^−1^). Furthermore, an increase in exopolysaccharide production was detected when cells were treated with 3.0 mg L^−1^l-lysine, indicating a resistance strategy that involves a metabolic process in cyanobacterial cells.

## Conflicts of interest

There are no conflicts to declare.

## Supplementary Material
